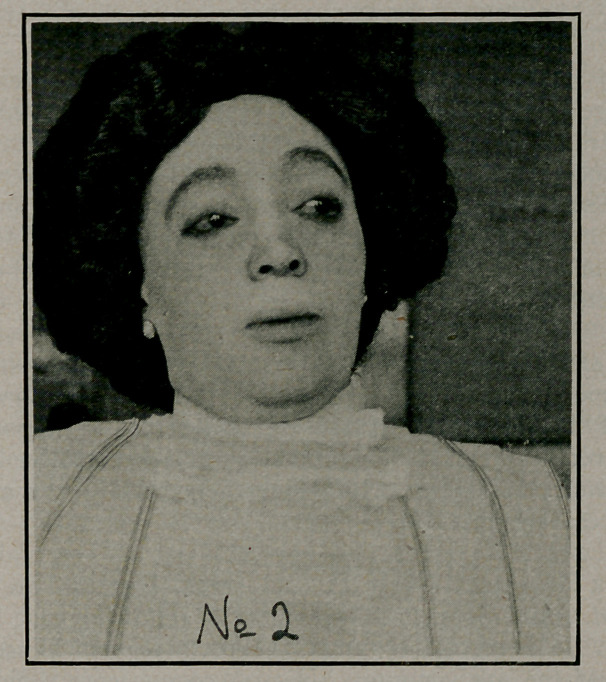# Ochronosis

**Published:** 1913-01

**Authors:** 


Ochronosis. Beddard and Plumtre, *Quarterly Jour. of Med.*, page 505, 1912, report a case in a man of 73 who had used car-

No 1

No 2

bolized oil for forty years for leg ulcers. He died of pneumonia. About 30 cases have been reported, 14 in connection with alkaptonuria, 9 with phenol, 7 not stated.

## Figures and Tables

**No 1 f1:**
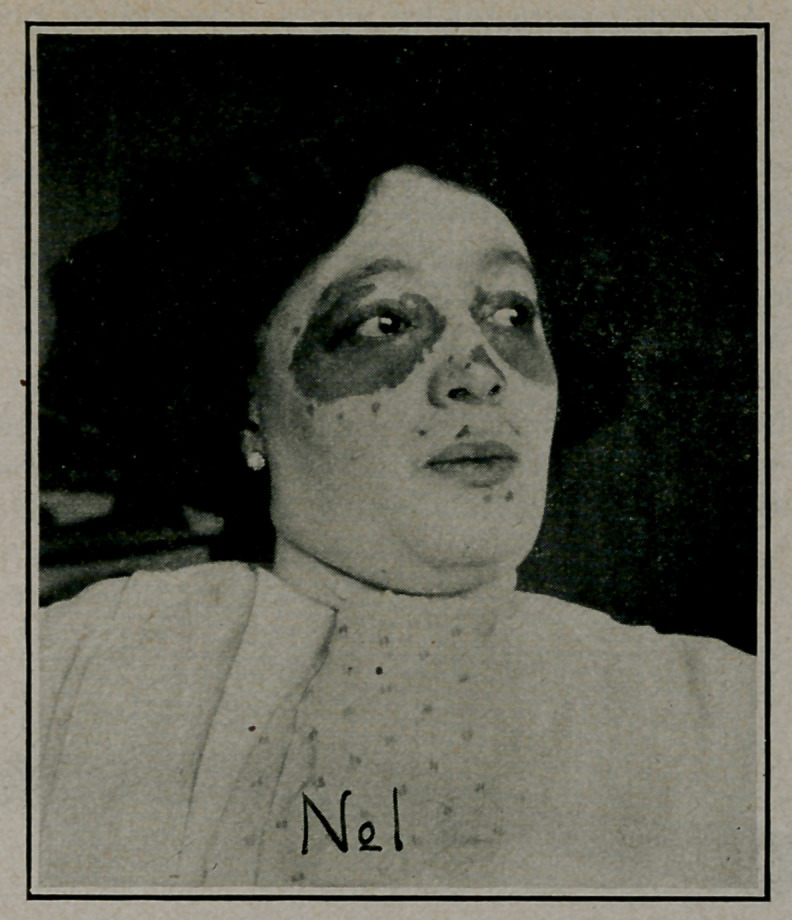


**No 2 f2:**